# Spatial Heterogeneity of Intratumoral Microbiota and Its Roles in Tumor–Microbiota Interactions and Therapeutic Implications

**DOI:** 10.3390/pathogens15070687

**Published:** 2026-06-30

**Authors:** Li Li, Xiaoqian Shi, Mingyang Liu, Tongzhen Xu, Yinan Chen, Ranjiaxi Wang, Qiyue Zhang, Dan Li

**Affiliations:** 1 State Key Laboratory of Molecular Oncology, National Cancer Center/National Clinical Research Center for Cancer/Cancer Hospital, Chinese Academy of Medical Sciences and Peking Union Medical College, Beijing 100021, China; lili150341@163.com (L.L.); shi18377936351@163.com (X.S.);; 2 Laboratory Animal Center, National Cancer Center/National Clinical Research Center for Cancer/Cancer Hospital, Chinese Academy of Medical Sciences and Peking Union Medical College, Beijing 100021, China

**Keywords:** intratumoral microbiota, spatial heterogeneity, tumor–microbe interactions

## Abstract

The intratumoral microbiota has emerged as a critical component of the tumor microenvironment (TME), with accumulating evidence indicating that its biological functions are influenced not only by microbial composition but also by their spatial organization within tumor tissues. This review summarizes the historical development and potential origins of intratumoral microbiota, and elaborates on the concept and biological significance of spatial heterogeneity. Based on recurrent spatial distribution patterns reported across different tumor types, we propose a conceptual framework comprising several putative spatial niches, including hypoxic/necrotic, immune-enriched, stromal-associated, invasive/metastatic, and intracellular niches. We further discuss the potential mechanisms contributing to the establishment and maintenance of spatial heterogeneity. The clinical significance of spatial microbial signatures is critically evaluated, alongside a comprehensive overview of spatial analytical methodologies, ranging from in situ hybridization and immunology-based approaches to emerging spatial omics and multi-omics integration strategies. Finally, we address key challenges and limitations, including contamination control, causal inference, barriers to clinical translation, and the underexplored spatial dimensions of the intratumoral mycobiome and virome. By synthesizing current knowledge and identifying critical gaps, this review aims to provide a conceptual and methodological framework for advancing spatially resolved investigations of intratumoral microbiota and facilitating their potential translational applications in precision oncology.

## 1. Introduction

The tumor microenvironment (TME) is a complex ecosystem. It consists of neoplastic, immune, and stromal cells, along with the extracellular matrix and diverse signaling factors. These components collectively drive tumor initiation, progression, and metastasis [1]. With the continued advancement of human microbiome research, intratumoral microbiota have been found to be widely present across various solid tumors and are increasingly recognized as an important component of TME studies [2]. Historically, microbial signals detected in tumor tissues were often dismissed as exogenous contamination [3]. This concern is especially relevant for low-biomass samples, where contaminating DNA can significantly skew the analytical results [4].

However, advancements in multi-omics technologies and rigorous decontamination protocols have shifted this perspective. Compelling evidence now confirms that tumor tissues harbor stable and biologically relevant microbial communities, including bacteria, fungi, and viruses [5,6,7]. Beyond their mere presence, emerging evidence indicates that these microbes are not uniformly distributed. Instead, they exhibit distinct spatial heterogeneity, often forming organized “micro-niches” in specific tumor regions [8]. This spatial organization significantly influences tumor–microbiota interactions, thereby modulating local immune responses and metabolic activities [9,10]. Although intratumoral microbiota include bacteria, fungi, and viruses, the current understanding of spatial heterogeneity is primarily derived from studies of bacteria. Accordingly, this review summarizes existing evidence on the spatial distribution of intratumoral bacterial communities, explores its impact on the TME, and discusses its potential implications for precision oncology.

## 2. Historical Progression of Intratumoral Microbiota Research Within the TME

Research on intratumoral microbiota has undergone a critical conceptual shift from the “contamination hypothesis” to the “taxonomic presence”. Although microbial presence in human tumors was documented over a century ago, their low biomass led early researchers to dismiss these observations as experimental artifacts or exogenous contamination. It was not until 2012 that Castellarin et al. [11] and Kostic et al. [12] independently reported a significant enrichment of *Fusobacterium nucleatum* (*F. nucleatum*) in colorectal cancer (CRC) tissues. Their findings provided the first robust evidence that specific microbes are associated with tumorigenesis.

Subsequent research shifted the focus from mere identification to functional characterization. It is now well-established that intratumoral bacteria modulate tumor progression, immune landscapes, and therapeutic efficacy. For example, specific bacteria can enzymatically degrade the chemotherapeutic drug gemcitabine, thereby inducing drug resistance [13]. Furthermore, studies have demonstrated that the composition of the microbiota significantly influences the host response to immune checkpoint inhibitors (ICIs), marking a critical milestone in understanding the microbiota’s role in clinical oncology [14,15]. These findings marked a pivotal transition in the field from merely establishing microbial presence to elucidating functional roles.

**Figure 1:**
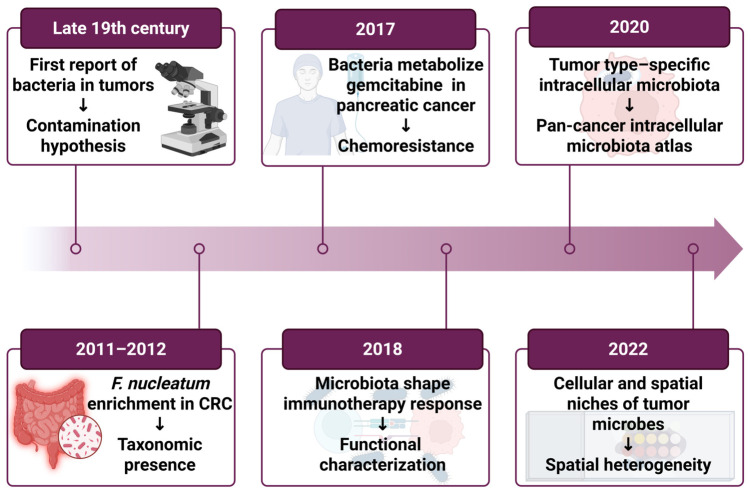
Historical progression in intratumoral microbiota research. Created in BioRender. Li, L. (2026) https://BioRender.com/gwtrxpz.

Building on this foundation, researchers have gradually recognized that focusing solely on microbial composition and abundance is insufficient to fully explain their biological significance. Moreover, the spatial distribution of microbiota is also crucial for their functional performance. Given the pronounced spatial heterogeneity of tumor tissues, intratumoral microbiota also exhibit non-random spatial distribution patterns. In 2020, Nejman et al. [2] analyzed 1526 tumor and adjacent samples across seven cancer types, confirming the existence of intratumoral microbiota and revealing their tumor type-specific distribution. Notably, their work also highlighted that the majority of these bacteria reside intracellularly within both cancer and immune cells. In 2022, leveraging advanced technologies such as spatial transcriptomics (e.g., InvadeSeq) and in situ imaging, Galeano Niño et al. [8] demonstrated that microbes occupy specific spatial niches. These microbial “micro-niches” are closely aligned with the localized immune and metabolic states of the TME. This discovery marked the beginning of a new era focused on the spatial heterogeneity of the intratumoral microbiota and its implications for precision medicine (Figure 1).

## 3. Origins of Intratumoral Microbiota

Although accumulating evidence has demonstrated that intratumoral microbiota are widely present across multiple solid tumors and can influence tumor initiation, progression, and therapeutic response through modulation of the tumor microenvironment [16,17], the precise routes by which these microorganisms enter and persist within the TME remain incompletely understood. It is generally accepted that intratumoral microbiota are primarily derived from the host’s commensal microbial communities. These microbes may migrate to tumor tissues through multiple biological routes, eventually achieving selective colonization and expansion within specific ecological niches. Among them, the oral and gut microbiota represent the two most important microbial reservoirs [18,19] and are closely associated with the development and progression of multiple gastrointestinal malignancies.

Current evidence suggests that intratumoral microbiota may originate from three major routes. First, translocation due to the disruption of mucosal barriers is considered one of the most important routes of microbial entry. In organs directly exposed to the external environment, such as the gastrointestinal, respiratory, and urogenital tracts, resident mucosal microbiota may invade tumor tissues during carcinogenesis due to compromised epithelial barrier integrity and enhanced local inflammatory responses. For instance, gastrointestinal microbes may penetrate damaged epithelial barriers and enter tumor tissues [20], while esophageal and lung cancers may be influenced by microbiota derived from the adjacent oropharyngeal cavity or upper respiratory tract [21,22]. This concept is supported by studies demonstrating bacterial infiltration across compromised epithelial barriers in colorectal and other epithelial malignancies [23]. Second, microbial migration from adjacent tissues or organs represents another potential route. It has been reported that the microbial communities in breast cancer tissues exhibit similarities to those in adjacent normal tissues [2], suggesting that peritumoral healthy tissues may serve as a reservoir for intratumoral microbiota. This notion is consistent with emerging evidence that tissue-adjacent microbial niches can seed tumor-associated microbial populations [24]. Third, dissemination via blood or lymphatic circulation may represent a route for distant microbial dissemination. Representative oral bacteria, such as *F. nucleatum* and *Porphyromonas gingivalis*, have been detected in colorectal, pancreatic, esophageal, and head and neck squamous cell carcinoma tissues [25,26,27,28], supporting the hypothesis of hematogenous dissemination.

## 4. Concept and Biological Significance of Spatial Heterogeneity in the Intratumoral Microbiota

The entry of microorganisms into tumor tissues does not necessarily imply their stable persistence within the tumor microenvironment. In fact, most microbes that enter the bloodstream or lymphatic circulation are rapidly eliminated by the host immune system, and only a small fraction are able to survive and establish stable colonization within tumor tissues [29]. Therefore, the unique ecological conditions provided by the tumor microenvironment constitute a critical prerequisite for the establishment of intratumoral microbiota. Accumulating evidence indicates that microorganisms within tumors exhibit highly heterogeneous and spatially organized distribution patterns rather than random dispersion throughout the tumor tissues [30]. Different tumor regions may harbor distinct microbial communities, and such spatial heterogeneity is closely associated with local metabolic status, immune environment, and tissue architecture, thereby potentially affecting tumor progression and treatment response. For example, in gastric cancer (GC), the intratumoral microbiota exhibits distinct vertical spatial heterogeneity across different anatomical regions of the stomach. Oral-associated taxa, including *Veillonella parvula*, *Streptococcus oralis*, and *Prevotella intermedia*, are preferentially enriched in the upper third, whereas *Helicobacter pylori* is more frequently detected in the lower third and appears to occupy a central position within regional microbial interaction networks [31]. These observations highlight the presence of region-specific microbial communities within tumors and support the notion that spatial microbial heterogeneity may participate in shaping distinct local tumor microenvironments. In addition, intratumoral microbiota are often organized within specialized micro-niches closely associated with epithelial and immune cell functions [8]. Similarly, in glioma and brain metastases samples, intracellular bacterial 16S rRNA and lipopolysaccharide (LPS) signals have been detected in tumor cells, immune cells and stromal cells [32]. Taken together, accumulating evidence suggests that intratumoral microbiota may serve as important regulators of tumor spatial architecture and localized cellular functions.

Biologically, the spatial heterogeneity of the intratumoral microbiota has emerged as an important component of the tumor immune microenvironment. Microbial enrichment or depletion in different regions may contribute to the formation of distinct ecological niches, thereby influencing tumor progression and therapeutic responsiveness [33]. In murine pancreatic cancer models, T cell-rich and T cell-poor regions have been reported to harbor distinct bacterial communities, further supporting the existence of spatially coordinated microbiota–immune interactions within tumors [34]. In addition to immune modulation, the spatial organization of intratumoral microbiota may also be associated with localized metabolic heterogeneity and metabolite distribution patterns [35].

Therefore, spatial heterogeneity is not merely a descriptive feature. It serves as a vital bridge linking host–microbe interactions with tumor progression, immune evasion, and drug response. A comprehensive understanding of these spatial patterns and their interactions with host cells is essential. Such insights will provide the theoretical foundation for developing precise, microbiome-based diagnostic tools and targeted therapies, thereby advancing the goals of precision oncology.

## 5. Spatial Distribution Patterns of the Intratumoral Microbiota

Tumor spatial heterogeneity highlights functional differences among distinct regions within a single tumor and holds important biological and clinical significance. With advances in spatial profiling technologies, recent studies have revealed functional niches within the TME characterized by unique cellular organization patterns and molecular signatures [36]. Similar to TME niches composed of immune or stromal cells, intratumoral microbiota are not uniformly distributed throughout tumor tissues but instead tend to be enriched in specific regions such as hypoxic and necrotic areas, invasive fronts, stromal compartments, and immune cell-rich zones, thereby forming distinct microbial aggregation patterns with diverse biological functions [35,37]. Beyond the tissue level, this heterogeneity extends to cellular and subcellular scales. Bacteria often localize within specific cell types, such as tumor cells or immune cells, and exhibit spatial co-localization with specific cellular populations [8]. Furthermore, the functional state of a microbe can be profoundly altered by its immediate environment. Specifically, the same species may perform different biological roles depending on whether it resides in the extracellular matrix or within the intracellular compartment [38].

**Figure 2:**
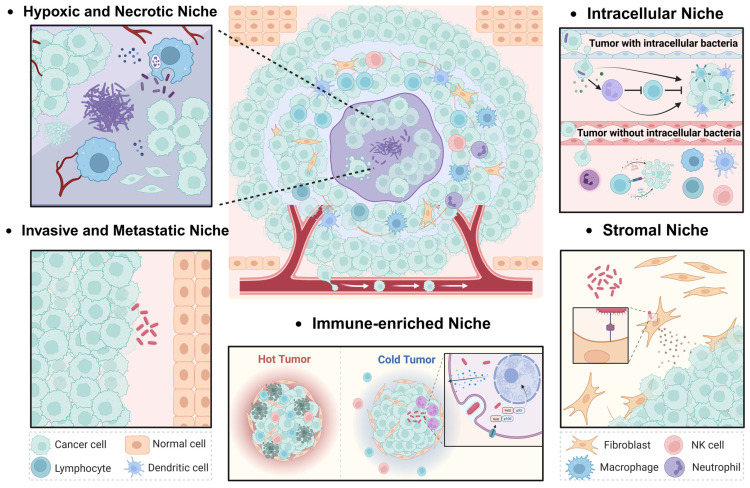
Proposed conceptual framework of intratumoral microbial spatial heterogeneity. Created in BioRender. Li, L. (2026) https://BioRender.com/ht1flcd.

Due to the relatively limited number of spatial studies on intratumoral microbiota, current understanding of their spatial niches is largely based on recurrent patterns of microbial enrichment across different studies, and a unified, widely accepted classification framework has yet to be established [39]. Therefore, in this review, representative spatial patterns are summarized based on host microenvironmental features including hypoxia/necrosis, immune infiltration, stromal activation, and invasive front, together with reported distributions of intratumoral microbiota in the existing literature. It should be emphasized that the niche categories discussed herein represent a conceptual framework based on current evidence, aiming to organize and interpret the spatial heterogeneity of intratumoral microbiota, rather than a standardized classification system. Based on this framework, we summarize five types of intratumoral microbial spatial niches with distinct functional characteristics (Figure 2) and discuss their potential interactions with the local microenvironment as well as their implications for tumor biological behavior.

### 5.1. Hypoxic and Necrotic Niche

Hypoxia and necrosis are among the most characteristic spatial features of the microenvironment in solid tumors and are typically identified using markers such as HIF-1α and CA9 expression [40], as well as histologically defined necrotic regions. Spatial transcriptomics and in situ spatial-profiling technologies in tumor tissues from patients with CRC have further revealed that certain bacterial populations are preferentially associated with areas characterized by low Ki-67 expression, reduced vascular density, and immunosuppressive features [8]. These observations collectively support the notion that microbial spatial distribution is closely linked to local immune and metabolic states within the tumor microenvironment. Consistently, a mouse model study examining the relationship between bacteria and tumor vasculature reported that large *Salmonella* colonies mainly accumulated in poorly vascularized tumor regions [41].

Additional studies in oral squamous cell carcinoma (OSCC) and CRC have shown that intratumoral bacteria are frequently localized within necrotic tumor regions, where neighboring cancer cells often exhibit reduced cellular density, proliferative activity, and transcriptional activity [37]. Similar spatial patterns have also been reported in tumor samples from patients with metastatic pancreatic ductal adenocarcinoma (PDAC), in which chronic hypoxia and pronounced intratumoral heterogeneity contribute to the formation of micronecrotic niches. Within these regions, bacterial components have been implicated in modulating tumor-associated macrophage activity, accompanied by enhanced PD-L1 expression and epithelial–mesenchymal transition (EMT) related features in pancreatic cancer cells [42]. Hypoxia-associated metabolic reprogramming may shape microbial colonization and bacterial functional states within tumors [43].

Collectively, current evidence suggests that hypoxic and necrotic tumor regions may provide unique ecological conditions that facilitate microbial colonization and host–microbe interactions. Bacteria residing in these regions may be potentially linked to tumor progression and therapeutic response through inflammatory signaling, immune suppression, and EMT-related pathways.

### 5.2. Immune-Enriched Niche

Another important spatial distribution pattern of intratumoral microbiota is their enrichment in regions of immune cell infiltration, particularly those characterized by the accumulation of CD8^+^ T cells, tumor-associated macrophages, neutrophils, and myeloid-derived suppressor cells [44,45,46,47]. Such spatial organization may contribute to the formation of adjacent immune-active (“hot”) and immunosuppressive (“cold”) niches within tumors [48]. Several studies have demonstrated spatial colocalization between intratumoral microbiota and these immune components. In a PDAC mouse model, the microbiota has been associated with TLR-related signaling, M2 macrophage polarization, and myeloid-derived suppressor cell (MDSC) accumulation, thereby contributing to an immunosuppressive microenvironment [17]. Similarly, in the GC mouse model, *F. nucleatum* has been implicated in IL-17/NF-κB/RelB signaling and tumor-associated neutrophil enrichment linked to immune evasion [49].

While T-cell-rich niches often harbor distinct bacterial communities compared to T-cell-poor regions, the depletion of these bacteria can fundamentally alter B-cell and myeloid phenotypes [34]. Furthermore, integrated spatial analyses using single-cell proteomics and 16S fluorescence in situ hybridization (FISH) in patient tissues demonstrated that MDSCs preferentially cluster around bacterial colonization sites in multifocal hepatocellular carcinoma, accompanied by reduced CD8^+^ T-cell infiltration [50]. Collectively, current evidence suggests a close spatial association between intratumoral microbiota and the local immune microenvironment, and microbial colonization in immune cell-enriched regions may contribute to the establishment of distinct immune states.

### 5.3. Invasive and Metastatic Niche

The tumor invasive front is a critical spatial region where tumor cells, stromal cells, and immune components undergo dynamic interactions. It is typically characterized by high proliferative activity, vascular remodeling, and alterations in the immune microenvironment, and can be identified based on histological features such as Ki-67 expression, vascular density, and the invasive boundary [36]. In recent years, several studies have reported that certain microorganisms tend to be enriched at the tumor invasive front [51] or at the interface between tumor and normal tissue. Increasing evidence suggests that microbiota localized within these regions may be associated with tumor invasion, progression, and metastatic dissemination. In breast cancer, *F. nucleatum* is enriched in cell-dense regions and has been linked to MAPK-related signaling and enhanced migratory phenotypes [52]. Similarly, in CRC, *F. nucleatum* is more likely to be enriched at the invasive tumor front and its abundance increases with tumor progression [12,53]. In pancreatic cancer, intratumoral *F. nucleatum* has also been associated with CXCL1-CXCR2-related signaling involved in tumor progression [54]. In addition, *Pseudoalteromonas elyakovii* has been identified in breast cancer tissues and appears to increase with disease progression, potentially contributing to stromal remodeling and tumor migration [55].

Distinct microbial spatial patterns have also been observed in metastatic lesions. In CRC liver metastases, bacteria tend to colonize metastatic lesions and areas adjacent to SOX9^+^ proliferative tumor cells [23]. Furthermore, tumor-resident intracellular microbiota have been implicated in enhancing the mechanical stress resistance and metastatic colonization capacity of circulating breast cancer cells [29]. Overall, invasive and metastatic niches appear to harbor distinct microbial communities. These microbial communities exhibit a close spatial association with tumor cell plasticity, local immune states, and metastasis-related biological processes; however, the mechanisms underlying their formation, as well as whether they directly contribute to tumor invasion and metastasis, remain to be fully elucidated and experimentally validated.

### 5.4. Stromal Niche

The tumor stroma is a key component of the tumor microenvironment, primarily composed of cancer-associated fibroblasts (CAFs), extracellular matrix (ECM), blood vessels, and immune cells [56]. Stromal activation is typically accompanied by CAF enrichment and ECM remodeling, which can be identified using CAF markers such as α-SMA and FAP, as well as histological features such as collagen deposition [57]. Studies have shown that regions enriched in CAFs or characterized by active stromal remodeling represent another important niche for intratumoral microbial colonization. In CRC mouse models, *F. nucleatum* has been reported to localize within CAF populations and is associated with inflammatory CAF-related phenotypes characterized by increased cytokine production and reactive oxygen species (ROS) accumulation [58]. Meanwhile, in ESCC, enrichment of *F. nucleatum* is closely associated with increased CAF infiltration and an immune-excluded phenotype, suggesting that it may promote CAF activation and stromal remodeling through inflammatory signaling pathways such as NF-κB, thereby facilitating tumor progression [59]. Therefore, the tumor stroma may also constitute a specific spatial niche for intratumoral microbial colonization and persistent survival. Dynamic interactions between microbes, CAFs, and the ECM may further amplify inflammatory responses, promote tissue remodeling and fibrosis, and ultimately shape a local microenvironment that favors tumor growth and immune evasion.

### 5.5. Intracellular Niche

Spatial heterogeneity of intratumoral microbiota is evident not only at the tissue level, but also at the subcellular level of microbial localization. Bacteria can reside either in the extracellular microenvironment or invade and persist within host cells over prolonged periods, and these distinct localization patterns may produce very different biological effects. In lung cancer, bacterial burden has been reported to be higher in malignant cells than in surrounding stromal or immune compartments and is associated with oncogenic signaling activity [60]. Moreover, recent studies have shown that the same bacterium can exert two distinct modes of action depending on whether it is intracellular or extracellular. For instance, intracellular bacteria within tumor cells have been linked to activation of the cGAS-STING-IL-17B axis and pro-metastatic neutrophil polarization, whereas extracellular localization of the same strain may be associated with antitumor neutrophil responses [38]. These findings highlight intracellular localization as an additional layer of spatial heterogeneity that may influence microbiota–host interactions and tumor progression.

These spatial niches are not entirely independent but may undergo dynamic transitions in response to tumor progression, changes in immune status, and therapeutic interventions. Collectively, current evidence supports the concept of the niche-specific spatial organization of intratumoral microbiota. However, much of the available evidence remains associative, and direct causal relationships are often inferred from preclinical models or descriptive spatial analyses rather than longitudinal functional studies. Future integration of high-resolution spatial multi-omics with experimental validation will be required to establish causal mechanisms underlying these spatial associations. Overall, intratumoral microbiota appear to exhibit multilayered spatial organization across distinct tumor niches, highlighting their potential roles in tumor heterogeneity, immune regulation, and clinical translation.

## 6. Mechanistic Basis of Spatial Heterogeneity Formation

An increasing number of studies have shown that the spatial distribution of intratumoral microbiota within tumor tissues is not random, but is dynamically shaped by differences among spatial niches within the TME. Distinct tumor regions exhibit marked variations in oxygen tension, metabolic gradients, vascularization, and immunosurveillance [61]. These regional microenvironmental features can selectively promote or restrict the colonization, survival, and expansion of specific microorganisms, thereby generating microbial enrichment patterns within defined spatial ranges. The spatial arrangement of these microbes, coupled with their interactions with the host, reciprocally reshapes the local TME [30]. While microorganisms may infiltrate tumor tissues via hematogenous dissemination or retrograde ductal migration [23], their subsequent spatial patterning is primarily dictated by local ecological selection.

### 6.1. Drivers of TME Heterogeneity

The physical, metabolic, and immunological heterogeneity within the TME is a central factor driving the spatial stratification of intratumoral microbiota. Aberrant angiogenesis and rapid tumor proliferation generate pronounced gradients of oxygen, pH, nutrients, and metabolites across tumor regions. Consequently, hypoxic, acidic, and necrotic cores coexist with relatively oxygenated and nutrient-rich invasive fronts or perivascular niches [62]. Such spatial metabolic heterogeneity can provide differential survival conditions for different microorganisms. For example, hypoxic and acidic environments may be more suitable for facultative anaerobes and obligate anaerobes, whereas the large amounts of lipids, nucleotides, and protein degradation products released from necrotic regions may serve as alternative nutrient sources for specific microorganisms [63]. In PDAC, local pH and oxygen levels have also been implicated in bacterial colonization patterns following retrograde migration through pancreatic ducts [64].

Beyond metabolic constraints, tissue architecture and local immune states further shape microbial spatial distribution. Epithelial integrity, extracellular matrix remodeling, vascular permeability, and immune cell infiltration within tumor tissues all vary considerably across regions and may jointly determine microbial entry and persistent colonization. Furthermore, molecular features of tumor cells facilitate selective recruitment. For example, *F. nucleatum* utilizes its Fap2 adhesin to bind Gal-GalNAc overexpressed on CRC cells, mediating site-specific enrichment [65]. Conversely, in regions rich in activated T cells, IFN signaling, or antibacterial immune activity, microorganisms may face stronger immune clearance pressure and thus fail to establish stable colonization. Consistently, *F. nucleatum* abundance in CRC has been reported to inversely correlate with CD3^+^ T-cell density [66], further supporting a close association between local immune states and microbial spatial heterogeneity.

### 6.2. Community Interactions Among Microorganisms

Inter-microbial interactions further refine the spatial architecture of the tumor microbiota. Beyond focusing on the spatial localization of bacteria, studies have further expanded to the tumor mycobiome. One study characterized fungal distribution across 35 cancer types, revealing synergistic interactions between bacteria and fungi and their collective potential as prognostic biomarkers [67]. Although direct studies on intratumoral microbial interactions remain limited, classical microecological theory suggests that intratumoral microbes may influence each other’s spatial distribution through mechanisms such as metabolic complementarity, nutrient competition, and quorum sensing [68]. Microorganisms may alter local oxygen tension or metabolic conditions by secreting metabolites, thereby promoting or inhibiting the colonization and expansion of other microbial taxa [69].

Such cooperative or competitive interactions at the community level may further enhance the stability of specific taxa within local micro-niches and ultimately form spatially clustered regions with distinct functional properties. For example, *F. nucleatum* and *Peptostreptococcus anaerobius* have been reported to co-enrich in recurrent colorectal cancer tissues and form dual-species biofilms through RadD-mediated aggregation [70]. This interaction appears to be influenced by local arginine metabolism, further suggesting a link between metabolic conditions and microbial spatial organization.

The formation of spatial heterogeneity in intratumoral microbiota is not a one-way process determined solely by the TME. Instead, it represents a bidirectional and dynamic process. Tumor-associated hypoxia, immune suppression, and metabolic heterogeneity may impose selective pressures on microbial colonization, whereas resident microorganisms may in turn influence local immune and metabolic states. Current evidence suggests that the spatial heterogeneity of intratumoral microbiota may be the result of long-term dynamic interactions between the TME and microbial communities, although the precise causal mechanisms remain to be elucidated.

## 7. Clinical Significance

Intratumoral microbiota and their metabolites profoundly influence immune function within the TME. These microbes act through multiple mechanisms, including functioning as stimulatory antigens, secreting cytokines, and participating in metabolic pathways, thereby inducing local immune responses [71]. The diversity of microbial communities within the TME leads to highly heterogeneous immune responses, ranging from immune activation to immune suppression. These diverse immune responses may promote both antitumor and protumor effects [72]. Therefore, understanding the spatial heterogeneity of intratumoral microbiota may not only help reveal the organizational principles of the tumor ecosystem, but also provide a new biological basis for precision oncology.

Spatial heterogeneity of intratumoral microbiota may also be a key reason for inconsistent findings across studies. Given that clinical biopsies typically capture only a fraction of the tumor volume and that microbial composition, abundance, and functional states vary significantly across different regions, single-site sampling is inherently susceptible to sampling bias. This undermines the stability and reproducibility of diagnostic outputs. Parallel challenges have been documented in tuberculosis research: even within a single necrotic lesion, the core harbors a higher density of *Mycobacterium tuberculosis* in a more metabolically and transcriptionally active state compared to the periphery [43]. This phenomenon is likely amplified in the intratumoral context, where microbial populations are characterized by low biomass and focal distribution. Therefore, future research should prioritize multi-region sampling or spatial-omics frameworks to enhance the clinical interpretability and consistency of microbiome data.

The spatial heterogeneity of intra-tumor microbiota may serve as a biomarker for prediction and prognosis. Intratumoral heterogeneity often manifests as a mosaic of “hot” and “cold” regions. Evidence from PDAC suggests that the presence of “cold” subpopulations can systemically impair antitumor immunity across the entire tumor landscape [73,74]. Because these immune niches are spatially heterogeneous, bulk microbiome profiling may obscure clinically relevant microbial patterns. Mouse models have effectively recapitulated the human spatial overlap between immune cells and microbes, revealing that T-cell-rich and T-cell-poor regions harbor distinct bacterial consortia associated with active versus quiescent phenotypes [34]. These findings suggest that spatial microbial features may serve as superior prognostic and predictive biomarkers compared to aggregate microbial abundance [75,76].

Moreover, the spatial heterogeneity of intratumoral microbiota may provide new strategies for microbiota-targeted therapy. For instance, taking advantage of the natural tropism of anaerobic bacteria for hypoxic regions, engineered bacterial platforms have been developed to selectively deliver chemotherapeutic agents into poorly vascularized tumor areas, thereby enhancing intratumoral drug accumulation [77]. With the continued development of engineered bacteria [78], probiotics [79], phage therapy, and locally delivered interventions based on microbial delivery systems [80], microbial regulation targeted to specific spatial niches may represent a new direction in cancer therapy. Compared with conventional strategies that globally modulate the microbiome, therapeutic approaches guided by microbial spatial organization may enable more precise targeting of functionally distinct tumor niches, thereby reducing off-target effects and improving local therapeutic efficacy. However, the clinical translation of these microbiota-targeted interventions necessitates careful consideration of their safety profiles. Engineered bacterial therapies require the robust attenuation of virulence, bio-containment strategies such as inducible suicide circuits, and rigorous assessment of immunogenicity to mitigate risks of systemic infection and chronic inflammation [81]. Meanwhile, although phage therapy and microbial delivery systems are generally well-tolerated in local applications, their systemic safety and long-term effects remain challenging issues that warrant further validation in rigorously controlled clinical trials [82,83].

## 8. Technical Methods for Spatial Analysis of Intratumoral Microbiota

Because intratumoral microbiota are typically low biomass, their detection and spatial resolution have long posed major challenges. In addition to traditional methods for microbial detection, identification, and culture, a variety of technologies based on in situ detection, spatial omics, and multi-omics integration have been developed, providing important tools for identifying the presence, distribution, and diversity of intratumoral microbiota [84]. These advances have provided key tools for investigating spatial interplay between microbial communities and host cellular architecture.

### 8.1. In Situ Hybridization-Based Techniques

In situ hybridization techniques allow for the direct visualization of microbial localization while preserving the integrity of the tissue architecture. FISH remains a cornerstone of this approach. This technique typically uses fluorescent probes targeting bacterial 16S rRNA to identify bacteria with high sensitivity and specificity [85]. FISH-based studies have confirmed that bacteria in various tumors are not randomly distributed but tend to enrich in specific intratumoral regions [52,86,87].

However, classical FISH often suffers from weak signals when detecting low-abundance microorganisms because of insufficient cell permeability and limited nucleic acid copy number. To circumvent these challenges, RNAscope technology has been adapted for microbial detection. By employing a unique “Z-probe” design to amplify signals while rigorously suppressing background noise, RNAscope enables single-molecule visualization in formalin-fixed paraffin-embedded (FFPE) tissues [88]. Therefore, this technique has been used to detect bacterial RNA within tissues as well as host–microbe interaction-related transcripts, and has been successfully applied to reveal the heterogeneous spatial distribution of *F. nucleatum* in CRC and OSCC [8].

### 8.2. Immunology-Based Methods

Immunohistochemistry (IHC) and immunofluorescence (IF) are based on the specific binding between antigens and antibodies and can be used to detect microbial components and host cell markers in tissues. IHC generates color through chemical reactions, whereas IF uses fluorescent labels for the qualitative and quantitative detection of antigens in tissues. These techniques have been instrumental in identifying bacterial LPS and lipoteichoic acid (LTA) within esophageal squamous cell carcinoma tissues [89]. Another study found the colocalization of bacteria and LPS in necrotic regions of PDAC tissues, suggesting that the local necrotic microenvironment may facilitate the enrichment of specific bacteria [42].

Beyond two-dimensional tissue section analysis, three-dimensional imaging combined with tissue clearing has also begun to be applied in tumor microbiome research. This approach facilitates the observation of microbial–tumor interactions across larger spatial volumes, as evidenced by the visualization of LPS signals within the complex 3D architecture of human gliomas [90]. Furthermore, advances in multiplex immunofluorescence, imaging mass cytometry, and Co-detection by Indexing (CODEX) have enabled the simultaneous analysis of multiple immune cell, stromal cell, and microbe-related markers in the same tissue section. These immunology-based methods not only validate microbial presence, but also reveal spatial interactions between microbial communities and local immune niches.

### 8.3. Spatial Omics and Multi-Omics Integration Approaches

In recent years, the rapid development of spatial omics technologies have significantly advanced research on intratumoral microbiota spatial heterogeneity. Spatial transcriptomics, spatial metabolomics, and spatial proteomics can systematically analyze the molecular characteristics of local regions while preserving tissue spatial information, providing new opportunities to study the relationship between intratumoral microbiota and local immune niches.

Currently, microbe–host functional links are often inferred by integrating microbial mapping with host spatial transcriptomics datasets. In terms of microbial identification, the PathSeq pipeline provides a computational framework for the unbiased extraction of microbial sequences from high-throughput host data [91,92]. Building upon this foundation, the team extended this work by developing PathSeq-T2T, an innovative bioinformatics pipeline that leverages the complete telomere-to-telomere human reference genome (T2T-CHM13) to enable refined host sequence depletion from tumor whole-genome sequencing data. By achieving near-complete elimination of host background, this method demonstrates substantially improved performance compared with previous approaches [93].

Next-generation spatial transcriptomic platforms have further improved the capacity for the co-detection of the host and microbes. For example, technologies such as Stereo-Seq V2 can simultaneously monitor dynamic changes in host and microbial transcriptomes at relatively high spatial resolution [94], opening new research avenues for elucidating host-microbe interaction mechanisms. Similarly, Spatial host-microbiome sequencing (SHM-Seq) utilizes dual-capture strategies (RNA and 16S rDNA) to correlate specific microbial consortia with host gene expression programs [95]. High-resolution platforms like CosMx [32] and MERFISH [96] further allow researchers to resolve the transcriptomic state of cells adjacent to microorganisms at single-cell resolution.

Complementing these genomic insights, spatial metabolomics can characterize metabolite patterns in tumor tissues and thus complement transcriptomic analyses. This technique can detect microbial behavior-related metabolic markers and establish links with specific TMEs. For instance, the integration of spatial metabolomics (MALDI-FTICR imaging) with 16S rRNA sequencing revealed that CoPEC in right-sided CRC can form a high-glycerophospholipid microenvironment locally, accompanied by reduced CD8^+^ T-cell infiltration [97]. Therefore, spatial metabolomics can help reveal microbiota-associated metabolic niches and their immunoregulatory roles. The development of spatial proteomics has likewise promoted research on intratumoral microbiota spatial heterogeneity. Technologies such as DSP, CODEX, MIBI, and IMC provide high-dimensional spatial protein analysis.

Integrated workflows like IN-DEPTH, combining CODEX-based proteomics with ST and advanced cross-correlation algorithms, have elucidated how viruses like EBV remodel the TME landscape [98]. Such spatial multi-omics integration enables the construction of a more refined spatial ecological map and reveals complex relationships among microbial aggregation sites, immune cell distribution, and metabolic gradients.

### 8.4. Complementary Applications and Methodological Integration

Based on the methodological characteristics described above, different technical approaches exhibit distinct and complementary applications in validating the spatial distribution of microorganisms. Spatial transcriptomics, when integrated with in situ techniques such as FISH or RNAscope, enables a complementary integration of transcript detection and single-cell localization, thereby providing a more robust basis for inferring spatial correlations. Multiplex immunofluorescence and imaging mass cytometry can further establish multidimensional associations between microorganisms and the local microenvironment; however, the reliability of such evidence is constrained by antibody specificity. In contrast, single-channel in situ hybridization or immunohistochemistry allows for intuitive visualization of the anatomical localization of microorganisms, yet it is limited in its ability to simultaneously capture host responses or functional states, resulting in a limited interpretative resolution of spatial information. Bioinformatic strategies that infer microbial distribution from host transcriptomic datasets (e.g., PathSeq) offer preliminary insights into spatial heterogeneity; nevertheless, these inferences require validation by in situ methods to confirm the true spatial relationships between microorganisms and host cells. Moreover, 16S rRNA gene sequencing, due to its limited taxonomic resolution and lack of subcellular localization capability, primarily serves a screening role in spatial heterogeneity studies, with its findings necessitating cross-validation by high-resolution in situ techniques.

Overall, spatial omics and multi-omics integration technologies are driving research on intratumoral microbiota from asking “whether microbes are present” toward understanding “how microbes participate in local spatial ecological regulation”. However, no single technology platform currently achieves high spatial resolution, high throughput, high sensitivity, and low background interference simultaneously (Table 1). Therefore, future development is more likely to rely on integrated multi-platform strategies, integrating in situ hybridization, spatial transcriptomics, and multi-omics analyses to systematically dissect the spatial heterogeneity and biological functions of intratumoral microbiota across different scales.

## 9. Challenges and Limitations

Despite substantial methodological advances in the study of intratumoral microbial heterogeneity, this field still faces multiple challenges and limitations, and recognizing and addressing these limitations can contribute to understanding the intratumoral mycobiome and virome and their contribution to cancer.

### 9.1. Sources and Control of Contamination

Given the extremely low biomass of intratumoral microbiota, contamination control remains a central challenge in this field [99]. Sources of contamination span multiple stages, including experimental handling, reagents and consumables, and computational analysis. These primarily include contaminant DNA and sample cross-contamination [4]. Contaminant DNA may originate from airborne microorganisms within sampling environments, microbes on operators’ skin surfaces, residual DNA in DNA extraction reagents and laboratory consumables, as well as environmental microbial intrusion during FFPE processing [100]. Cross-contamination represents another major challenge in microbiome research. DNA or amplified products from adjacent wells or tubes may transfer between samples, generating false-positive signals and batch effects. Aerosol dissemination during PCR amplification and leakage between wells can also lead to cross-contamination. In addition, non-target index tags may infiltrate adjacent sample wells during sequencing library preparation, leading to “index hopping” and consequent misassignment of sequencing reads across samples [4]. Computational pipelines may further introduce artifactual signals, for example, by misclassifying human-derived reads as bacterial sequences or by relying on contaminated reference databases.

To mitigate these issues, Fierer et al. [101] proposed an international consensus guideline for contamination prevention in low-biomass microbiome studies, providing a comprehensive workflow spanning experimental design and data analysis. During sampling, instruments should undergo ethanol sterilization, nucleic acid degradation treatment, autoclaving, or UV-C irradiation, and negative controls such as extraction blanks and no-template amplification controls should be included. In laboratory practice, strict physical separation of pre- and post-DNA extraction areas and pre- and post-PCR areas is required, together with the use of certified DNA-free consumables and contamination testing for each reagent batch [4,101]. Given that experimental measures cannot fully eliminate contamination, computational decontamination approaches serve as an essential complement. Methods such as decontam, microDecon, SourceTracker, Squeegee, and SCRuB can be used to identify and remove reads derived from exogenous contaminants, thereby improving data reliability through post hoc correction [102,103,104,105,106].

### 9.2. Investigation of Causal Mechanisms

At present, most studies on the spatial heterogeneity of intratumoral microbiota remain correlative, and the causal mechanisms by which microorganisms participate in tumor evolution, immunomodulation, and therapeutic sensitivity in a spatial context remain largely unresolved. Whether intratumoral microbiota are merely passive colonizers occupying pre-existing niches or active organizers shaping spatial architectures within the tumor microenvironment—this fundamental question remains to be addressed through integrated multi-omics approaches.

Specifically, germ-free animal models combined with spatial profiling can be used to test whether exogenously introduced microbes are capable of reconstructing spatial ecological patterns. Species-specific antibiotics or bacteriophage-based targeting strategies, coupled with longitudinal monitoring of the tumor microenvironment, may help determine whether microbial depletion can reshape local immune and metabolic landscapes [107,108]. Fluorescently labeled or bioluminescent bacterial strains delivered via oral gavage or intravenous injection can be employed to dynamically track bacterial tropism toward specific tumor regions and the temporal establishment of spatial niches [109]. Furthermore, longitudinal sampling along the cancer continuum—from precancerous lesions, carcinoma in situ, to invasive cancer—is critical for defining stage-specific microbial colonization dynamics and their temporal association with tumor progression [110]. Future studies should integrate spatial multi-omics with these causality-oriented experimental frameworks to establish robust mechanistic links between microbial spatial organization and tumor biology.

### 9.3. Clinical Translation

Although accumulating evidence suggests an association between intratumoral microbial spatial heterogeneity and tumor biology, its clinical translation remains in an early stage. A key prerequisite for clinical adoption is whether spatial microbial features can provide independent predictive value beyond conventional clinicopathological parameters such as tumor stage, histological subtype, immune infiltration, and genomic alterations.

First, standardization of tissue sampling and processing remains a major bottleneck. Current studies exhibit substantial methodological heterogeneity in sample type (fresh-frozen vs. FFPE), section thickness, DNA extraction protocols, spatial analysis platforms, and contamination control strategies. These inconsistencies introduce inter-institutional batch effects and significantly compromise reproducibility [111,112]. Second, there is a lack of validated clinical thresholds: the extent of taxonomic enrichment within a given spatial niche that constitutes clinical relevance remains undefined, and such thresholds are likely tumor-type and site specific. In the absence of clearly defined cutoffs, spatial microbial features risk being arbitrarily interpreted, potentially leading to misleading clinical decisions. Third, validation cohorts remain insufficient, as most existing evidence derives from single-center retrospective studies with limited sample sizes, and their generalizability requires confirmation in prospective multicenter cohorts. Fourth, spatial microbial profiling involves complex multi-omics workflows, and its cost-effectiveness remains unclear, further limiting clinical implementation. Future efforts should prioritize the standardization of sampling and analytical pipelines, systematic validation of clinical thresholds, and the establishment of large-scale prospective multicenter cohorts to facilitate the translation of spatial microbial features from basic research into clinical decision-making tools.

### 9.4. Spatial Challenges of the Mycobiome and Virome

In addition, although 16S rRNA gene sequencing and FISH have provided preliminary spatial distribution maps, high-resolution spatial localization of fungal and viral components remains technically challenging and relatively underexplored [7,113]. Compared with bacteria, the study of fungal and viral spatial heterogeneity faces additional limitations, including their relatively low biomass [114], high susceptibility to contamination, and the difficulty of simultaneously capturing these components together with host cellular architecture in situ [115]. Furthermore, unlike bacteria, viruses lack a universally conserved phylogenetic marker analogous to the 16S rRNA gene [116], while fungal identification relies primarily on ITS regions that are limited by incomplete and inconsistently annotated reference databases [117].

Consequently, the spatial organization and ecological roles of the intratumoral mycobiome and virome remain poorly defined, and their interactions with bacterial communities and host cells are still largely unexplored. This limited understanding of non-bacterial components also hampers the establishment of a comprehensive framework for characterizing intratumoral microbial spatial niches. Constructing a universally applicable spatial microbial atlas, elucidating inter-kingdom interactions within tumor microbiomes (bacteria, fungi, viruses), and enabling cross-study data integration and standardized analytical frameworks remains a critical future direction.

## 10. Conclusions

The synergy of high-resolution in situ detection, high-throughput sequencing, and spatial omics technologies has fundamentally reshaped our understanding of TME [118]. The spatial heterogeneity of intratumoral microbiota may provide a new avenue for precision oncology. This paradigm shift emphasizes that microbial function is governed not merely by their presence, but more critically, by their spatial biogeography. As demonstrated by the landmark study of Galeano Niño [8], microbial spatial enrichment patterns are intricately coupled with local immunological status and tissue architecture. This study has shifted the field from simple microbial correlation toward a mechanistic understanding of how microorganisms participate in the spatial ecological regulation of tumors. Compared with traditional analyses based on overall microbial abundance, the spatial relationships among microorganisms, tumor cells, immune cells, and the local metabolic environment may better reflect their true functional states and biological significance.

In conclusion, cancer is recognized as a dynamic, multi-kingdom spatial ecosystem where tumor cells, host immune components, and microbial communities coexist in a delicate balance. A deeper understanding of these spatial host–microbe interactions will not only help redefine the organizational principles of the TME, but also provide a new theoretical basis and intervention strategy for precision medicine and individualized therapy.

## Figures and Tables

**Table 1:** Technical methods for the spatial analysis of intratumoral microbiota.

Category	Method	Application
In situ hybridization- based techniques	FISH	Direct visualization of microbial localization
	RNAscope	Detect bacterial RNA within tissues and host–microbe interaction-related transcripts
Immunology-based methods	IHC, IF	Qualitative and quantitative detection
	3D imaging combined with tissue clearing	Observation of microbial–tumor interactions across larger spatial volumes
	mIF, IMC, CODEX	Reveal spatial interactions between microbes and local immune niches
Spatial transcriptomics	PathSeq, PathSeq-T2T	Extraction of microbial sequences from high-throughput host data
	Stereo-Seq V2, SHM-Seq, CosMx, MERFISH	Co-detection of host and microbes
Spatial metabolomics	MALDI-FTICR imaging	Reveal microbiota-associated metabolic niches
Spatial proteomics	DSP, CODEX, MIBI, IMC	Provide high-dimensional spatial protein analysis
Integrated workflows	IN-DEPTH	Integration of spatial single-cell proteomics and spatial transcriptomics

## Data Availability

Not applicable.

## Abbreviations

The following abbreviations are used in this manuscript:

TME	Tumor microenvironment
*Fusobacterium nucleatum*	*F. nucleatum*
ICIs	Immune checkpoint inhibitors
GC	Gastric cancer
LPS	Lipopolysaccharide
OSCC	Oral squamous cell carcinoma
CRC	Colorectal cancer
PDAC	Pancreatic ductal adenocarcinoma
EMT	Epithelial–mesenchymal transition
MDSC	Myeloid-derived suppressor cell
FISH	Fluorescence in situ hybridization
CAF	Cancer-associated fibroblast
ROS	Reactive oxygen species
FFPE	Formalin-fixed paraffin-embedded
IHC	Immunohistochemistry
IF	Immunofluorescence
LTA	Lipoteichoic acid
CODEX	Co-detection by Indexing
SHM-Seq	Spatial host-microbiome sequencing

## References

[1] de Visser K.E., Joyce J.A. (2023). The evolving tumor microenvironment: From cancer initiation to metastatic outgrowth. Cancer Cell.

[2] Nejman D., Livyatan I., Fuks G., Gavert N., Zwang Y., Geller L.T., Rotter-Maskowitz A., Weiser R., Mallel G., Gigi E. (2020). The human tumor microbiome is composed of tumor type-specific intracellular bacteria. Science.

[3] Salter S.J., Cox M.J., Turek E.M., Calus S.T., Cookson W.O., Moffatt M.F., Turner P., Parkhill J., Loman N.J., Walker A.W. (2014). Reagent and laboratory contamination can critically impact sequence-based microbiome analyses. BMC Biol..

[4] Eisenhofer R., Minich J.J., Marotz C., Cooper A., Knight R., Weyrich L.S. (2019). Contamination in Low Microbial Biomass Microbiome Studies: Issues and Recommendations. Trends Microbiol..

[5] Dohlman A.B., Arguijo Mendoza D., Ding S., Gao M., Dressman H., Iliev I.D., Lipkin S.M., Shen X. (2021). The cancer microbiome atlas: A pan-cancer comparative analysis to distinguish tissue-resident microbiota from contaminants. Cell Host Microbe.

[6] Qiao H., Tan X.-R., Li H., Li J.-Y., Chen X.-Z., Li Y.-Q., Li W.-F., Tang L.-L., Zhou G.-Q., Zhang Y. (2022). Association of Intratumoral Microbiota with Prognosis in Patients with Nasopharyngeal Carcinoma from 2 Hospitals in China. JAMA Oncol..

[7] Mou W., Deng Z., Zhu L., Jiang A., Lin A., Xu L., Deng G., Huang H., Guo Z., Zhu B. (2025). Intratumoral mycobiome heterogeneity influences the tumor microenvironment and immunotherapy outcomes in renal cell carcinoma. Sci. Adv..

[8] Galeano Niño J.L., Wu H., LaCourse K.D., Kempchinsky A.G., Baryiames A., Barber B., Futran N., Houlton J., Sather C., Sicinska E. (2022). Effect of the intratumoral microbiota on spatial and cellular heterogeneity in cancer. Nature.

[9] Liu N.-N., Yi C.-X., Wei L.-Q., Zhou J.-A., Jiang T., Hu C.-C., Wang L., Wang Y.-Y., Zou Y., Zhao Y.K. (2023). The intratumor mycobiome promotes lung cancer progression via myeloid-derived suppressor cells. Cancer Cell.

[10] Zhou J., Hu Z., Wang L., Hu Q., Chen Z., Lin T., Zhou R., Cai Y., Wu Z., Zhang Z. (2024). Tumor-colonized Streptococcus mutans metabolically reprograms tumor microenvironment and promotes oral squamous cell carcinoma. Microbiome.

[11] Kostic A.D., Chun E., Robertson L., Glickman J.N., Gallini C.A., Michaud M., Clancy T.E., Chung D.C., Lochhead P., Hold G.L. (2013). Fusobacterium nucleatum potentiates intestinal tumorigenesis and modulates the tumor-immune microenvironment. Cell Host Microbe.

[12] Castellarin M., Warren R.L., Freeman J.D., Dreolini L., Krzywinski M., Strauss J., Barnes R., Watson P., Allen-Vercoe E., Moore R.A. (2012). Fusobacterium nucleatum infection is prevalent in human colorectal carcinoma. Genome Res..

[13] Geller L.T., Barzily-Rokni M., Danino T., Jonas O.H., Shental N., Nejman D., Gavert N., Zwang Y., Cooper Z.A., Shee K. (2017). Potential role of intratumor bacteria in mediating tumor resistance to the chemotherapeutic drug gemcitabine. Science.

[14] Anker J.F., Naseem A.F., Mok H., Schaeffer A.J., Abdulkadir S.A., Thumbikat P. (2018). Multi-faceted immunomodulatory and tissue-tropic clinical bacterial isolate potentiates prostate cancer immunotherapy. Nat. Commun..

[15] Xin G., Schauder D.M., Jing W., Jiang A., Joshi N.S., Johnson B., Cui W. (2017). Pathogen boosted adoptive cell transfer immunotherapy to treat solid tumors. Proc. Natl. Acad. Sci. USA.

[16] Li X., Wu D., Li Q., Gu J., Gao W., Zhu X., Yin W., Zhu R., Zhu L., Jiao N. (2024). Host-microbiota interactions contributing to the heterogeneous tumor microenvironment in colorectal cancer. Physiol. Genom..

[17] Pushalkar S., Hundeyin M., Daley D., Zambirinis C.P., Kurz E., Mishra A., Mohan N., Aykut B., Usyk M., Torres L.E. (2018). The Pancreatic Cancer Microbiome Promotes Oncogenesis by Induction of Innate and Adaptive Immune Suppression. Cancer Discov..

[18] Irfan M., Delgado R.Z.R., Frias-Lopez J. (2020). The Oral Microbiome and Cancer. Front. Immunol..

[19] Zhou C.-B., Zhou Y.-L., Fang J.-Y. (2021). Gut Microbiota in Cancer Immune Response and Immunotherapy. Trends Cancer.

[20] Hold G.L., Hansen R. (2019). Impact of the Gastrointestinal Microbiome in Health and Disease: Co-evolution with the Host Immune System. Curr. Top. Microbiol. Immunol..

[21] Khan F.H., Bhat B.A., Sheikh B.A., Tariq L., Padmanabhan R., Verma J.P., Shukla A.C., Dowlati A., Abbas A. (2022). Microbiome dysbiosis and epigenetic modulations in lung cancer: From pathogenesis to therapy. Semin. Cancer Biol..

[22] Gao P., Yuan H., Mei Z., Yin X., Zeng Y., Liu Z., Yang X., Xue J., Liu Z., Jiang Y. (2025). The comprehensive oral microbiome landscape unveils its interplay with poor oral health in esophageal squamous cell carcinoma risk. Cell Rep. Med..

[23] Bertocchi A., Carloni S., Ravenda P.S., Bertalot G., Spadoni I., Lo Cascio A., Gandini S., Lizier M., Braga D., Asnicar F. (2021). Gut vascular barrier impairment leads to intestinal bacteria dissemination and colorectal cancer metastasis to liver. Cancer Cell.

[24] Sipos A., Ujlaki G., Mikó E., Maka E., Szabó J., Uray K., Krasznai Z., Bai P. (2021). The role of the microbiome in ovarian cancer: Mechanistic insights into oncobiosis and to bacterial metabolite signaling. Mol. Med..

[25] Saba E., Farhat M., Daoud A., Khashan A., Forkush E., Menahem N.H., Makkawi H., Pandi K., Angabo S., Kawasaki H. (2024). Oral bacteria accelerate pancreatic cancer development in mice. Gut.

[26] Tan Q., Ma X., Yang B., Liu Y., Xie Y., Wang X., Yuan W., Ma J. (2022). Periodontitis pathogen Porphyromonas gingivalis promotes pancreatic tumorigenesis via neutrophil elastase from tumor-associated neutrophils. Gut Microbes.

[27] Zang W., Geng F., Liu J., Wang Z., Zhang S., Li Y., Lu Z., Pan Y. (2025). Porphyromonas gingivalis potentiates stem-like properties of oral squamous cell carcinoma by modulating SCD1-dependent lipid synthesis via NOD1/KLF5 axis. Int. J. Oral Sci..

[28] Pignatelli P., Nuccio F., Piattelli A., Curia M.C. (2023). The Role of Fusobacterium nucleatum in Oral and Colorectal Carcinogenesis. Microorganisms.

[29] Fu A., Yao B., Dong T., Chen Y., Yao J., Liu Y., Li H., Bai H., Liu X., Zhang Y. (2022). Tumor-resident intracellular microbiota promotes metastatic colonization in breast cancer. Cell.

[30] Lin A., Xiong M., Jiang A., Huang L., Wong H.Z.H., Feng S., Zhang C., Li Y., Chen L., Chi H. (2025). The microbiome in cancer. iMeta.

[31] Lei L., Zhao L.-Y., Cheng R., Zhang H., Xia M., Chen X.-L., Kudriashov V., Liu K., Zhang W.-H., Jiang H. (2024). Distinct oral-associated gastric microbiota and Helicobacter pylori communities for spatial microbial heterogeneity in gastric cancer. mSystems.

[32] Morad G., Damania A.V., Melendez B., Singh B.B., Veguilla F.J., Soto R.A., Hoballah Y.M., Sahasrabhojane P.V., Wong M.C., Ahmed M.M. (2025). Microbial signals in primary and metastatic brain tumors. Nat. Med..

[33] Battaglia T.W., Mimpen I.L., Traets J.J.H., van Hoeck A., Zeverijn L.J., Geurts B.S., de Wit G.F., Noë M., Hofland I., Vos J.L. (2024). A pan-cancer analysis of the microbiome in metastatic cancer. Cell.

[34] Li Y., Chang R.B., Stone M.L., Delman D., Markowitz K., Xue Y., Coho H., Herrera V.M., Li J.H., Zhang L. (2024). Multimodal immune phenotyping reveals microbial-T cell interactions that shape pancreatic cancer. Cell Rep. Med..

[35] Yao Y., Zhu Y., Chen K., Chen J., Li Y., Li D., Wei P. (2026). Microbiota in cancer: Current understandings and future perspectives. Signal Transduct. Target. Ther..

[36] Liu Y., Dai Y., Wang L. (2026). Spatial omics at the forefront: Emerging technologies, analytical innovations, and clinical applications. Cancer Cell.

[37] Galeano Niño J.L., Ponath F., Ajisafe V.A., Becker C.R., Kempchinsky A.G., Zepeda-Rivera M.A., Gomez J.A., Wu H., Terrazas J.G., Bouzek H. (2026). Tumor-infiltrating bacteria disrupt cancer epithelial cell interactions and induce cell-cycle arrest. Cancer Cell.

[38] Yao B., Liu X., Ruan K., Fang X., Jiang C., Bian W., Guo Y., Zhu X., Shang Z., Hu T. (2026). Divergent tumor immunity determined by bacteria-cancer cell engagement. Cell.

[39] Liao K., Wen J., Liu Z., Zhang B., Zhang X., Fu Y., Zhang W., Hu H., Ai K., Zhu W. (2025). The role of intratumoral microbiome in the occurrence, proliferation, metastasis of colorectal cancer and its underlying therapeutic strategies. Ageing Res. Rev..

[40] Chen Z., Han F., Du Y., Shi H., Zhou W. (2023). Hypoxic microenvironment in cancer: Molecular mechanisms and therapeutic interventions. Signal Transduct. Target. Ther..

[41] Zhang M., Forbes N.S. (2015). Trg-deficient Salmonella colonize quiescent tumor regions by exclusively penetrating or proliferating. J. Control. Release Off. J. Control. Release Soc..

[42] Chen Q., Xu Y., Wang Y., Zheng S., Yao T., Qiu J., Qin H., Liang T. (2025). Bacteria and tumor debris induced pancreatic cancer progression via the NF-κB signaling pathway. Cancer Lett..

[43] Lavin R.C., Tan S. (2022). Spatial relationships of intra-lesion heterogeneity in Mycobacterium tuberculosis microenvironment, replication status, and drug efficacy. PLoS Pathog..

[44] Chai X., Wang J., Li H., Gao C., Li S., Wei C., Huang J., Tian Y., Yuan J., Lu J. (2023). Intratumor microbiome features reveal antitumor potentials of intrahepatic cholangiocarcinoma. Gut Microbes.

[45] Lam K.C., Araya R.E., Huang A., Chen Q., Di Modica M., Rodrigues R.R., Lopès A., Johnson S.B., Schwarz B., Bohrnsen E. (2021). Microbiota triggers STING-type I IFN-dependent monocyte reprogramming of the tumor microenvironment. Cell.

[46] Alam A., Levanduski E., Denz P., Villavicencio H.S., Bhatta M., Alhorebi L., Zhang Y., Gomez E.C., Morreale B., Senchanthisai S. (2022). Fungal mycobiome drives IL-33 secretion and type 2 immunity in pancreatic cancer. Cancer Cell.

[47] Silver N.L., Dai J., Kerr T.D., Altemus J., Garg R., Simmons H., Alban T., Noel-Romas L., Makarov V., Shih D.J.H. (2026). Intratumoral bacteria are immunosuppressive and promote immunotherapy resistance in head and neck squamous cell carcinoma. Nat. Cancer.

[48] Wu B., Zhang B., Li B., Wu H., Jiang M. (2024). Cold and hot tumors: From molecular mechanisms to targeted therapy. Signal Transduct. Target. Ther..

[49] Zhang T., Li Y., Zhai E., Zhao R., Qian Y., Huang Z., Liu Y., Zhao Z., Xu X., Liu J. (2025). Intratumoral Fusobacterium nucleatum Recruits Tumor-Associated Neutrophils to Promote Gastric Cancer Progression and Immune Evasion. Cancer Res..

[50] Lu Y., Xu L., Chen W., Liu W., Zhang Y., Zhou Q., Wang N., Zhang Y., Bai H., Xu S. (2025). Intrahepatic Microbial Heterogeneity in Multifocal Hepatocellular Carcinoma and Its Association with Host Genomic and Transcriptomic Alterations. Cancer Discov..

[51] Xu Y., Wang M.-C. (2025). The intratumoral microbiota in breast cancer: From basic research to clinical translation. Gut Microbes.

[52] Zhao F., An R., Ma Y., Yu S., Gao Y., Wang Y., Yu H., Xie X., Zhang J. (2025). Integrated spatial multi-omics profiling of Fusobacterium nucleatum in breast cancer unveils its role in tumour microenvironment modulation and cancer progression. Clin. Transl. Med..

[53] Yamamoto S., Kinugasa H., Hirai M., Terasawa H., Yasutomi E., Oka S., Ohmori M., Yamasaki Y., Inokuchi T., Harada K. (2021). Heterogeneous distribution of Fusobacterium nucleatum in the progression of colorectal cancer. J. Gastroenterol. Hepatol..

[54] Hayashi M., Ikenaga N., Nakata K., Luo H., Zhong P., Date S., Oyama K., Higashijima N., Kubo A., Iwamoto C. (2023). Intratumor Fusobacterium nucleatum promotes the progression of pancreatic cancer via the CXCL1-CXCR2 axis. Cancer Sci..

[55] Liu S., Pan Y., Zheng C., Zheng Q., Du Y., Zheng Y., Tang H., Liu X., Mou J., Zeng X. (2025). Tumor-colonizing Pseudoalteromonas elyakovii metabolically reprograms the tumor microenvironment and promotes breast ductal carcinoma. mBio.

[56] Lv Y., Duan T., Song J., Liu S., Zhou Z., Ba Y., Weng S., Zuo A., Xu H., Luo P. (2026). The Spatiotemporal Heterogeneity of Tumor-Associated Stromal Cells: Reprogramming Plasticity to Unlock Precision Cancer Immunotherapy. Cancer Commun..

[57] Cords L., de Souza N., Bodenmiller B. (2024). Classifying cancer-associated fibroblasts-The good, the bad, and the target. Cancer Cell.

[58] Karta J., Meyers M., Rodriguez F., Koncina E., Gilson C., Klein E., Gabola M., Benzarti M., Pérez Escriva P., Molina Tijeras J.A. (2025). Fusobacterium nucleatum interacts with cancer-associated fibroblasts to promote colorectal cancer. EMBO J..

[59] Ofuchi T., Hu Q., Omachi K., Kanemitsu K., Otsu H., Yonemura Y., Tobo T., Baba Y., Iwatsuki M., Mimori K. (2026). Intratumoral Fusobacterium nucleatum Drives Cancer-Associated Fibroblasts Enrichment and Immune Exclusion in Esophageal Squamous Cell Carcinoma. Ann. Gastroenterol. Surg..

[60] Wong-Rolle A., Dong Q., Zhu Y., Divakar P., Hor J.L., Kedei N., Wong M., Tillo D., Conner E.A., Rajan A. (2022). Spatial meta-transcriptomics reveal associations of intratumor bacteria burden with lung cancer cells showing a distinct oncogenic signature. J. Immunother. Cancer.

[61] Wang M., Yu F., Li P. (2023). Intratumor microbiota in cancer pathogenesis and immunity: From mechanisms of action to therapeutic opportunities. Front. Immunol..

[62] Wu C., Xu T., Zhang H., Hu Y., Jiao J., Qiu K., Gu J., Li W., Sun L. (2025). Hypoxia and immunometabolism in the tumor microenvironment: Insights into mechanisms and therapeutic potential. Cancer Lett..

[63] Walker S.P., Tangney M., Claesson M.J. (2020). Sequence-Based Characterization of Intratumoral Bacteria—A Guide to Best Practice. Front. Oncol..

[64] Shirai H., Ito C., Tsukada K. (2022). pH-taxis drives aerobic bacteria in duodenum to migrate into the pancreas with tumors. Sci. Rep..

[65] Abed J., Emgård J.E., Zamir G., Faroja M., Almogy G., Grenov A., Sol A., Naor R., Pikarsky E., Atlan K.A. (2016). Fap2 Mediates Fusobacterium nucleatum Colorectal Adenocarcinoma Enrichment by Binding to Tumor-Expressed Gal-GalNAc. Cell Host Microbe.

[66] Mima K., Sukawa Y., Nishihara R., Qian Z.R., Yamauchi M., Inamura K., Kim S.A., Masuda A., Nowak J.A., Nosho K. (2015). Fusobacterium nucleatum and T Cells in Colorectal Carcinoma. JAMA Oncol..

[67] Narunsky-Haziza L., Sepich-Poore G.D., Livyatan I., Asraf O., Martino C., Nejman D., Gavert N., Stajich J.E., Amit G., González A. (2022). Pan-cancer analyses reveal cancer-type-specific fungal ecologies and bacteriome interactions. Cell.

[68] Paniz M.I., Tina R.L., Farzaneh D., Farbod B., Nima R. (2025). Bacterial Quorum Sensing: A Double-Edged Sword in Cancer Development. Adv. Biol..

[69] Situ Y., Zhang P., Zhang C., Jiang A., Zhang N., Zhu L., Mou W., Liu Z., Wong H.Z.H., Zhang J. (2025). The metabolic dialogue between intratumoural microbes and cancer: Implications for immunotherapy. eBioMedicine.

[70] Zhang Y., Zhang B., Pang W., Gu W., Wang X., Yuan H., Fang S., Zhang J., Li X., Xing X. (2026). The interplay between *Peptostreptococcus* and *Fusobacterium* as novel signatures in colorectal cancer recurrence. Microbiome.

[71] Xu W., Zhou Y., Ding H., Ye M., Li S., Xu L., Jin X., Zhan Z., Song L., Zhang Y. (2025). PreTA-mediated metabolism of 5-fluorouracil by intratumoral Citrobacter freundii drives chemoresistance in pancreatic cancer. Cell Rep..

[72] Goto Y., Iwata S., Miyahara M., Miyako E. (2023). Discovery of Intratumoral Oncolytic Bacteria Toward Targeted Anticancer Theranostics. Adv. Sci..

[73] Li J., Byrne K.T., Yan F., Yamazoe T., Chen Z., Baslan T., Richman L.P., Lin J.H., Sun Y.H., Rech A.J. (2018). Tumor Cell-Intrinsic Factors Underlie Heterogeneity of Immune Cell Infiltration and Response to Immunotherapy. Immunity.

[74] Tanaka M., Lum L., Hu K.H., Chaudhary P., Hughes S., Ledezma-Soto C., Samad B., Superville D., Ng K., Chumber A. (2025). Tumor cell heterogeneity drives spatial organization of the intratumoral immune response. J. Exp. Med..

[75] Sun L., Ke X., Guan A., Jin B., Qu J., Wang Y., Xu X., Li C., Sun H., Xu H. (2023). Intratumoural microbiome can predict the prognosis of hepatocellular carcinoma after surgery. Clin. Transl. Med..

[76] Yuan Q., Sun Y., Zhang Y., Chen C., Bu C., Hua X., Sun L., Sun Y., Zhang Z., Feng Y. (2026). Intratumoral P. copri Reprograms MARCO+ Tumor-Associated Macrophages by Depleting Glycerophosphocholine to Drive Colorectal Cancer Progression. Cancer Res..

[77] Xiao S., Shi H., Zhang Y., Fan Y., Wang L., Xiang L., Liu Y., Zhao L., Fu S. (2022). Bacteria-driven hypoxia targeting delivery of chemotherapeutic drug proving outcome of breast cancer. J. Nanobiotechnol..

[78] Xu S., Zhang T., Song Y., Wang M., Wu R., Li M., Wang H., Xie X., Chen Q., Ma X. (2026). Sustained nitric oxide production by engineered *E. coli* remodels the tumor microenvironment and potentiates immunotherapy. Nat. Biotechnol..

[79] Li B., Liu G., Zhu Y., Chen L., Zhang N., Zhu X., Sun Y., Liu X., Liu W., Ma Y. (2026). Leveraging intratumoral probiotics for pancreatic cancer immunotherapy via xenophagy. Cell Host Microbe.

[80] Zou Z.-P., Wang X.-G., Shi X.-R., Sun S.-T., Mi J., Zhang X.-P., Yin B.-C., Zhou Y., Ye B.-C. (2025). Self-Adjusting Engineered Probiotic for Targeted Tumor Colonization and Local Therapeutics Delivery. Adv. Sci..

[81] Chen J., Chen J., Chen Y., Yuan W., Zhang J., Wang G., Dai Z. (2025). Engineering Bacteria as Living Therapeutics in Cancer Therapy. Adv. Sci..

[82] Ragothaman M., Yoo S.Y. (2023). Engineered Phage-Based Cancer Vaccines: Current Advances and Future Directions. Vaccines.

[83] Kaur T., Sharma D. (2024). Self-propelling bacteria-based magnetic nanoparticles (BacMags) for targeted magnetic hyperthermia therapy against hypoxic tumors. Nanoscale.

[84] Gui F., Zhang L., Xiao J., Zeng C. (2025). Decoding the role of intratumoral microbiota in gastric cancer. Biochim. Biophys. Acta Rev. Cancer.

[85] Gu J., Wang H., Zhang M., Xiong Y., Yang L., Ren B., Huang R. (2022). Application of Fluorescence In Situ Hybridization (FISH) in Oral Microbial Detection. Pathogens.

[86] Tao M., Wu T., Li S., Tan Y., Zhou X., Chen Y., Huang L., Wang W., Li S., Wang L. (2025). Intratumoral Collinsella aerofaciens exhibits antitumor activity in endometrial carcinoma through activation of the p53 signaling pathway. J. Transl. Med..

[87] Huang J.-H., Wang J., Chai X.-Q., Li Z.-C., Jiang Y.-H., Li J., Liu X., Fan J., Cai J.-B., Liu F. (2022). The Intratumoral Bacterial Metataxonomic Signature of Hepatocellular Carcinoma. Microbiol. Spectr..

[88] Wang F., Flanagan J., Su N., Wang L.-C., Bui S., Nielson A., Wu X., Vo H.-T., Ma X.-J., Luo Y. (2012). RNAscope: A novel in situ RNA analysis platform for formalin-fixed, paraffin-embedded tissues. J. Mol. Diagn. JMD.

[89] Wu H., Leng X., Liu Q., Mao T., Jiang T., Liu Y., Li F., Cao C., Fan J., Chen L. (2023). Intratumoral Microbiota Composition Regulates Chemoimmunotherapy Response in Esophageal Squamous Cell Carcinoma. Cancer Res..

[90] Zhao J., He D., Lai H.M., Xu Y., Luo Y., Li T., Liang J., Yang X., Guo L., Ke Y. (2022). Comprehensive histological imaging of native microbiota in human glioma. J. Biophotonics.

[91] Kostic A.D., Ojesina A.I., Pedamallu C.S., Jung J., Verhaak R.G., Getz G., Meyerson M. (2011). PathSeq: Software to identify or discover microbes by deep sequencing of human tissue. Nat. Biotechnol..

[92] Walker M.A., Pedamallu C.S., Ojesina A.I., Bullman S., Sharpe T., Whelan C.W., Meyerson M. (2018). GATK PathSeq: A customizable computational tool for the discovery and identification of microbial sequences in libraries from eukaryotic hosts. Bioinformatics.

[93] Dohlman A.B., Mjelle R., Wood H.M., Jiang K., Shumate A., Lee I., Piccinno G., Serna G., Yakubu A.R., Nuciforo P. (2026). Biodiversity and biogeography of the multi-kingdom cancer microbiome. Cell.

[94] Zhao Y., Li Y., He Y., Wu J., Liu Y., Li X., Li Z., Yuan Q., Li J., Zhang X. (2025). Stereo-seq V2: Spatial mapping of total RNA on FFPE sections with high resolution. Cell.

[95] Lötstedt B., Stražar M., Xavier R., Regev A., Vickovic S. (2024). Spatial host-microbiome sequencing reveals niches in the mouse gut. Nat. Biotechnol..

[96] Sarfatis A., Wang Y., Twumasi-Ankrah N., Moffitt J.R. (2025). Highly multiplexed spatial transcriptomics in bacteria. Science.

[97] de Oliveira Alves N., Dalmasso G., Nikitina D., Vaysse A., Ruez R., Ledoux L., Pedron T., Bergsten E., Boulard O., Autier L. (2024). The colibactin-producing Escherichia coli alters the tumor microenvironment to immunosuppressive lipid overload facilitating colorectal cancer progression and chemoresistance. Gut Microbes.

[98] Yiu S.P.T., Chang Y., Yeo Y.Y., Qiu H., Wu W., Michel H.A., Jin X., Huang R., Kure S., Parmelee L. (2026). Same-Slide Spatial Multi-Omics Integration with IN-DEPTH Reveals Tumor Virus-Linked Spatial Reorganization of the Tumor Microenvironment. Cancer Discov..

[99] Wang Z., Zhang T., Liu Y. (2025). Emerging technologies and current challenges in intratumoral microbiota research. Front. Cell. Infect. Microbiol..

[100] Weyrich L.S., Farrer A.G., Eisenhofer R., Arriola L.A., Young J., Selway C.A., Handsley-Davis M., Adler C.J., Breen J., Cooper A. (2019). Laboratory contamination over time during low-biomass sample analysis. Mol. Ecol. Resour..

[101] Fierer N., Leung P.M., Lappan R., Eisenhofer R., Ricci F., Holland S.I., Dragone N., Blackall L.L., Dong X., Dorador C. (2025). Guidelines for preventing and reporting contamination in low-biomass microbiome studies. Nat. Microbiol..

[102] Davis N.M., Proctor D.M., Holmes S.P., Relman D.A., Callahan B.J. (2018). Simple statistical identification and removal of contaminant sequences in marker-gene and metagenomics data. Microbiome.

[103] McKnight D.T., Huerlimann R., Bower D.S., Schwarzkopf L., Alford R.A., Zenger K.R. (2019). microDecon: A highly accurate read-subtraction tool for the post-sequencing removal of contamination in metabarcoding studies. Environ. DNA.

[104] Knights D., Kuczynski J., Charlson E.S., Zaneveld J., Mozer M.C., Collman R.G., Bushman F.D., Knight R., Kelley S.T. (2011). Bayesian community-wide culture-independent microbial source tracking. Nat. Methods.

[105] Liu Y., Elworth R.A.L., Jochum M.D., Aagaard K.M., Treangen T.J. (2022). De novo identification of microbial contaminants in low microbial biomass microbiomes with Squeegee. Nat. Commun..

[106] Austin G.I., Park H., Meydan Y., Seeram D., Sezin T., Lou Y.C., Firek B.A., Morowitz M.J., Banfield J.F., Christiano A.M. (2023). Contamination source modeling with SCRuB improves cancer phenotype prediction from microbiome data. Nat. Biotechnol..

[107] Bullman S., Pedamallu C.S., Sicinska E., Clancy T.E., Zhang X., Cai D., Neuberg D., Huang K., Guevara F., Nelson T. (2017). Analysis of Fusobacterium persistence and antibiotic response in colorectal cancer. Science.

[108] Dong X., Pan P., Zheng D.-W., Bao P., Zeng X., Zhang X.-Z. (2020). Bioinorganic hybrid bacteriophage for modulation of intestinal microbiota to remodel tumor-immune microenvironment against colorectal cancer. Sci. Adv..

[109] Parhi L., Alon-Maimon T., Sol A., Nejman D., Shhadeh A., Fainsod-Levi T., Yajuk O., Isaacson B., Abed J., Maalouf N. (2020). Breast cancer colonization by Fusobacterium nucleatum accelerates tumor growth and metastatic progression. Nat. Commun..

[110] Dejea C.M., Fathi P., Craig J.M., Boleij A., Taddese R., Geis A.L., Wu X., DeStefano Shields C.E., Hechenbleikner E.M., Huso D.L. (2018). Patients with familial adenomatous polyposis harbor colonic biofilms containing tumorigenic bacteria. Science.

[111] Sinha R., Abu-Ali G., Vogtmann E., Fodor A.A., Ren B., Amir A., Schwager E., Crabtree J., Ma S., Abnet C.C. (2017). Assessment of variation in microbial community amplicon sequencing by the Microbiome Quality Control (MBQC) project consortium. Nat. Biotechnol..

[112] Plummer J.T., Dezem F.S., Cook D.P., Park J., Zhang L., Liu Y., Marção M., DuBose H., Wani A., Wise K. (2025). Standardized metrics for assessment and reproducibility of imaging-based spatial transcriptomics datasets. Nat. Biotechnol..

[113] Yuan K., Xu H., Li S., Coker O.O., Liu W., Wang L., Zhang X., Yu J. (2025). Intraneoplastic fungal dysbiosis is associated with colorectal cancer progression and host gene mutation. eBioMedicine.

[114] Ajeh I.J., Ikukpla’si O.S.I. (2026). The non-bacterial oncobiome: The role of the mycobiome and virome in tumor plasticity. J. Egypt. Natl. Cancer Inst..

[115] Liu W., Pi Z., Liu N.-N., Mao W. (2024). Into the era of mycobiome-driven cancer research. Trends Cancer.

[116] Smith S.E., Huang W., Tiamani K., Unterer M., Khan Mirzaei M., Deng L. (2022). Emerging technologies in the study of the virome. Curr. Opin. Virol..

[117] Ding T., Liu C., Li Z. (2025). The mycobiome in human cancer: Analytical challenges, molecular mechanisms, and therapeutic implications. Mol. Cancer.

[118] Zhang N., Kandalai S., Zhou X., Hossain F., Zheng Q. (2023). Applying multi-omics toward tumor microbiome research. iMeta.

